# Shoulder Impingement Pain Syndrome: Pathophysiology, Diagnosis, and a Review of Current Treatment Strategies

**DOI:** 10.7759/cureus.92045

**Published:** 2025-09-11

**Authors:** Maitha Ibrahim Al Hammadi, Zakir Ali Shah, Rajesh Kumar Rathod, Mohamed Abdalla Seddik

**Affiliations:** 1 Trauma and Orthopedics, College of Medicine, University of Sharjah, Sharjah, ARE; 2 Trauma and Orthopaedics, Rashid Hospital, Dubai, ARE

**Keywords:** impingement syndrome, rotator cuff tendinopathy, shoulder impingement syndrome, “shoulder pain”, subacromial impingement syndrome

## Abstract

The shoulder is an intricate joint, capable of a wide range of movements for both daily activities and physical exercise. This intricate joint is susceptible to various injuries and conditions due to its design. Shoulder impingement syndrome (SIS), also clinically recognized as subacromial impingement syndrome (SAIS), is a prevalent source of shoulder discomfort, which is a spectrum of disorders such as rotator cuff tendinopathy, partial or complete tears, and inflammation of the subacromial bursa. SAIS can be caused by intrinsic causes, such as tendon degeneration, and extrinsic factors, such as structural abnormalities of the acromion, which contribute together to compress structures underneath the subacromial space. Initially, conservative management methods, such as organised physiotherapy and corticosteroid injections, are always advised to reduce pain, inflammation, and restore the range of motion of the shoulder. Surgical intervention, particularly acromioplasty, has been traditionally utilized whenever conservative treatment fails. In acromioplasty, a part of the acromion is shaved off to widen the subacromial space, thus releasing the pressure on the rotator cuff tendons and bursa. SAIS presentations make it harder to standardize treatments due to their diverse presentations, which emphasizes the importance of thorough, long-term research to improve diagnostic criteria and find the best ways to treat patients.

## Introduction and background

Epidemiology and risk factors

Subacromial impingement syndrome is a prevalent cause of shoulder pain, representing roughly 40% to 64% of all shoulder-related complaints in clinical settings [1]. In the Netherlands, the annual rate of rotator cuff tendinitis is thought to be between 3.2 and 4.2 per 1,000 people, while the annual rate of general shoulder pain is thought to be around 11.2 per 1,000 people. In the UK, almost half of the population will have shoulder pain at some point in their lives, and about 25% of these cases are caused by subacromial impingement syndrome (SAIS) [2].

Mechanism of impingement and past perspectives

SAIS is very common in people who perform repetitive overhead tasks, like swimmers, volleyball players, electricians, and hairdressers, because their arms are always raised. These repetitive tasks in these professionals put stress on rotator cuff tendons and subacromial bursa, leading to inflammation, a decrease in the subacromial space. and pain. SAIS occurs when the anatomical structures in the subacromial space (the space underneath the acromion and head of the humerus) develop inflammation and degeneration. This space, which is usually 1 to 1.5 cm wide, gets even smaller when the arm is 90° abducted and internally rotated [3], likelihood of compression. The humeral head can move upwards due to the loss of dynamic stability in the shoulder, leading to compression of the rotator cuff tendons and bursa underneath the acromion. In 1972, Dr. Charles Neer introduced the idea of mechanical impingement caused by bony spurs on the undersurface of the acromion and suggested acromioplasty as a way to treat [4].

Etiological theories and contributing factors

Despite advances in medical science, the precise etiology of SAIS is unclear and is a subject of debate between surgeons and researchers. This difficulty is due to the contribution of many factors, including intrinsic tendon degeneration and extrinsic compression. This uncertainty complicates the development of a unified theory of causation, prompting researchers to explore both biomechanical and histopathological evidence to better elucidate the primary drivers of SAIS and guide more targeted therapeutic approaches. There are two different theories regarding the development of Subacromial impingement. The intrinsic theory asserts that rotator cuff degeneration occurs before impingement, whereas the extrinsic theory contends that impingement results in tendon damage. Recent support for the intrinsic model highlights that inadequate vascularity in the supraspinatus tendon’s "critical zone" is a significant factor, particularly in older adults and individuals with diabetes or rheumatoid arthritis [5]. Histopathological results have found disorganized collagen and cellular architecture in the affected tendons, which signify unsuccessful healing efforts. On the other hand, the extrinsic theory says that the configuration of the acromion (flat, curved, or hooked) is the factor that leads to the narrowing of the space underneath the acromion. Acromion is classified into three types according to its anatomical and radiographic studies [6], each of which is believed to have some effect on narrowing and causing impingement. Certain factors that may exacerbate the situation include thoracic kyphosis, scapular dyskinesis, and weak musculature. External influences such as smoking, infections, and certain antibiotics are thought to contribute to these issues as well.

Treatment overview

In the past, acromioplasty was the usual treatment for SAIS when conservative treatments didn't work, like physiotherapy and steroid injections to relieve pain. This acromioplasty was widely used to relieve mechanical pressure on the rotator cuff and bursa, in which the acromion was shaved to increase the subacromial space. However, recent randomized controlled trials and systematic reviews indicate that its success is heavily dependent on postoperative rehabilitation. Non-surgical management, such as physiotherapy, ultrasound, and steroid injections, has been found effective in 60% of patients within two years, with overall success rates reaching between 70% and 90% [7]. These results suggest that a conservative approach should be prioritized, especially when there is no significant structural damage, and that surgical solutions may not provide better long-term outcomes when compared to rehabilitation programs.

## Review

Biomechanics of the shoulder and shoulder girdle joints

Dynamics of the Shoulder Joint

The glenohumeral joint, clinically known as the shoulder joint, is a very flexible synovial joint that allows three rotational movements (extension/flexion, adduction/abduction, and external/internal rotation) and three translational movements [8]. During active elevation of the arm in the plane of the scapula, which is about 30-40° in front of the frontal plane, the humerus rotates outward. This movement is crucial for allowing the greater tuberosity to pass through the coracoacromial arch and alleviates tension in the surrounding capsuloligamentous structures, facilitating a full elevation of the arm. During the early phase of arm lifting (30-60°), the humeral head ascends by roughly 1-3 mm. Beyond this angle, the head of the humerus remains predominantly centered within the glenoid cavity, exhibiting minimal vertical movement (less than 1 mm), allowing the joint to function as a typical ball-and-socket joint [9]. The initial superior translation is primarily ascribed to the upward pressure applied by the deltoid muscle, which is counterbalanced by the stabilising function of the rotator cuff muscles. The movement of the humeral head from anterior to posterior is less noticeable, but it plays a crucial role in maintaining the joint's stability and function.

In passive flexion, the humeral head can glide anteriorly from 2-5 mm, while in active flexion generally produces less than 1 mm of anterior glide, because the rotator cuff muscles stabilize the shoulder joint [10,11]. This translation plays a vital role in keeping the humeral head centred in the glenoid cavity, preventing stress on surrounding structures, thereby decreasing complications related to impingement. In healthy adults, the subacromial space is about 10-15 mm wide. 

As the arm is lifted, the available space decreases, causing the acromion to press against the underlying structures, such as the supraspinatus tendon and subacromial bursa, resulting in compression. Too much movement of the humeral head to the front or top can make compression worse, which could cause pain or damage to tissue [12] by disrupting the alignment of the joint and decreasing the available space. Clinically, this abnormal translation is observed in people with rotator cuff problems or scapular instability and is connected to pain during activities that involve raising the arms overhead. Diagnostic methods such as dynamic ultrasound or MRI can help in identifying these movements. In addition, rehabilitation programs such as targeted rotator cuff strengthening and scapular stabilization exercises are designed to restore normal humeral head positioning and reduce impingement risk. These additions aim to enhance the clinical integration of the biomechanical concepts presented.

Movement of the Shoulder Girdle Joint

The connection between the scapula and the thorax, referred to as the scapulothoracic joint, is a false anatomical joint; however, this serves as the functional link connecting the scapula and the thoracic cage. It enables five fundamental movements: three rotations (external rotation, upward rotation, and posterior tilt) and two translations (elevation/depression and protraction/retraction) [13]. 

Upward rotation occurs around an axis that runs from front to back, shifting the scapula's lower angle laterally and positioning the glenoid fossa appropriately to support the humeral head. External rotation occurs around a vertical axis, retracting the lateral edge of the scapula to better align the glenoid more toward the anterolateral position. Posterior tilt rotates the scapula around a medial-lateral axis, which lets the acromion tip back and clear the subacromial space during elevation.

There is a strong connection between scapular translations and clavicular motion at the sternoclavicular joint, for ensuring proper shoulder movement and function. The scapula's position is affected by the clavicle's elevation, depression, protraction, and retraction. The clavicle links the sternoclavicular and acromioclavicular joints, so its movement controls the position of the scapula. When the arm goes up, the clavicle usually goes up and back slightly, which puts the scapula higher and more to the back. Individuals with subacromial impingement syndrome frequently experience changes in scapular movement. Those affected show reduced posterior tilt and upward rotation, along with increased internal rotation (scapular winging) when compared to healthy shoulders. These alterations are often associated with muscular imbalances, including weakness in the serratus anterior and lower trapezius, fatigue in the infraspinatus and teres minor, and postural issues such as thoracic kyphosis and forward head posture are frequently documented in clinical and biomechanical research [14]. These alterations can interfere with scapular movement and the stability of the glenohumeral joint, leading to a reduction in subacromial space and increasing mechanical strain on the rotator cuff. Although these relationships are well established through observational studies, we acknowledge that high-quality comparative trials are limited. Further randomized studies are necessary to clarify causal connections and measure how these factors affect the progression of SAIS and the outcomes of rehabilitation.

Pathogenesis and etiological factors of SAIS

What is SAIS, and what are the factors that contribute to it? Initially, it was believed that issues with the rotator cuff were caused by the encroachment of the rotator cuff tendons against the undersurface of the acromion process, the ligament connecting the coracoid process and acromion, or the lower side of the acromioclavicular joint. SAIS now encompasses a range of disorders within the subacromial area, including tears involving rotator cuff tendons, tendinopathy of biceps or supraspinatus, calcific tendinitis, and inflammation of the subacromial bursa. SAIS initiates when the soft tissues in the subacromial space undergo compression, leading to inflammation and difficulties with movement. The rotator cuff tendons, which are crucial for stabilising and moving the shoulder, are especially prone to this kind of compression. SAIS is usually thought to be caused by intrinsic, extrinsic, or combined factors [15].

Intrinsic Mechanisms

 Intrinsic impingement is called when the rotator cuff tendons themselves start to break, often resulting from repetitive microtrauma, age-associated degeneration, or impaired vascular supply, especially in the “critical zone” of the supraspinatus tendon. These factors can cause tendinopathy, partial-thickness tears, or full-thickness ruptures over time [16].

Extrinsic Mechanisms

Extrinsic impingement occurs when surrounding anatomical structures compress the rotator cuff tendons from the outside. This includes: primary external impingement, which happens when the subacromial space gets smaller because of the shape of the acromion, osteophytes, or thickened coracoacromial ligaments [17].

Internal impingement occurs when the undersurface of the rotator cuff gets compressed between the posterior glenoid rim and the head of the humerus during extreme abduction and external rotation. This condition is frequently seen in athletes who engage in overhead throwing activities, such as baseball players. The acromiohumeral distance, measuring the gap between the acromion and the head of the humerus, is an important indicator of subacromial clearance. In healthy people, this distance is between 7 and 14 mm. A decrease to less than 7 mm, particularly at rest, correlates with a heightened risk of impingement and may indicate suboptimal surgical results [18].

Anatomical Factors

Some anatomical structures can make people more likely to develop SAIS, like the shape of the acromion, flat (type I), curved (type II), or hooked (type III), affecting the subacromial space. Type III (hooked) acromion exhibits the highest correlation with rotator cuff tears [14]. In addition, Coracoacromial ligament thickening and osteophytes in the acromioclavicular joint can reduce space and increase tendon compression. Although these structural characteristics are commonly seen in patients with SAIS, sensitivity and specificity data for acromion type as a diagnostic marker are inconsistent across studies, and current evidence suggests that anatomical findings alone should not guide treatment decisions. Surgical resection of portions of the coracoacromial arch has not consistently demonstrated superior outcomes compared to conservative management, reinforcing the multifactorial nature of SAIS and the need for individualized assessment.

Biomechanical Factors

Biomechanical Factors can contribute to the development of SAIS, which involve tightness in the posterior capsule of the glenohumeral joint and decreased strength of the rotator cuff muscles, both of which contribute to the onset of subacromial impingement syndrome by reducing subacromial clearance.

Tightness in the Shoulder Joint Capsule

The posterior capsule of the shoulder can change how the joint works when it is tight. Cadaveric studies have demonstrated that posterior capsular contraction induces superior and anterior translation of the humeral head during flexion, thereby diminishing the subacromial space and elevating the risk of impingement [19]. This condition can be clinically assessed through internal rotation deficits or horizontal adduction tests.

Dysfunction of the Muscles Associated With the Scapula

The serratus anterior and the lower trapezius are two of the most important scapular stabilisers for proper scapular motion. If these muscles are weak, the scapula will not rotate upward, tilt backward, or rotate outward as much, which decreases the subacromial space [20].

Thoracic Spine Alignment

It is believed that excessive thoracic kyphosis changes the position of the scapula at rest, which makes it tilt forward and makes it harder for the scapula to rotate up when the arm is raised, which leads to SAIS [21].

Imbalance in the Rotator Cuff Muscles

The supraspinatus, infraspinatus, teres minor, and subscapularis muscles collaborate to maintain the position of the humeral head within the glenoid fossa. Weakness or fatigue in these muscles permits the humeral head to ascend during elevation, thereby compressing the subacromial tissues [22].

Diagnosis of subacromial impingement syndrome and the role of clinical assessment

History of the Patient

Shoulder pain that becomes worse over time is a common sign of SAIS. It usually happens in the front and side of the shoulder, near the acromion. Activities that require reaching or grooming overhead often exacerbate the pain, which can spread down the upper arm. Experiencing pain at night is frequent, especially when resting on the affected side or when the arm is held up during sleep. Patients may report stiffness or weakness; however, these symptoms are frequently secondary to pain rather than structural damage [23].

Physical Examination

Several clinical tests can help clinicians diagnose SAIS clinically, but none of them are conclusive on their own. Neer's test is performed by keeping the arm internally rotated and passively flexing it forward, which causes pain in the front and side of the shoulder. It demonstrates a sensitivity of approximately 76% and a specificity of 36%. Similarly, the Hawkins-Kennedy test, which involves passively rotating the arm inwards while the shoulder is positioned at 90° of flexion, elicits pain and shows a sensitivity of approximately 80% and a specificity of 41%. To enhance diagnostic accuracy, the painful arc test, the empty can test (also known as the Jobe test), and the external rotation resistance test are frequently used in combination. When three or more tests yield positive results, the probability of Subacromial Impingement Syndrome (SAIS) increases significantly [24], with reported positive likelihood ratios ranging from 5.0 to 10.6 depending on the combination used. This multi-test approach enhances clinical confidence and supports more targeted management strategies.

The Neer injection test, which involves injecting an anaesthetic into the subacromial space, may help confirm the diagnosis if it relieves pain during provocative testing [25,26].

Differential diagnoses of subacromial impingement syndrome

There are a few shoulder conditions that need to be understood while making a differential diagnosis for SAIS.

Rotator cuff tear and long head of biceps tear are the clinical conditions, often reported with pain in the shoulder and reduced strength during activities. However, they can be differentiated from by the presence of persistent weakness despite the improvement of pain, which is less likely in SAIS.

Neurological problems like thoracic outlet syndrome, cervical radiculopathy, and brachial plexus injury present with clinical features of shoulder pain with radiation, alterations in sensation (such as tingling and numbness), and reduced muscle strength on the affected side. However, these can be differentiated from SAIS by the presence of weakness and paraesthesia; these features are not seen in SAIS.

Adhesive capsulitis and Calcific tendinitis present with global limitation of movements, difficulty with overhead/reaching movements, and can be differentiated from SAIS with persistent stiffness despite pain relief.

Arthritics clinical conditions, including acromioclavicular (AC) joint arthritis and glenohumeral arthritis, clinically reveal tenderness localized to the AC joint and can be differentiated from SAIS generalized discomfort with ongoing weakness and persistent stiffness in relation to pain.

Tears of the shoulder muscles, neurologic conditions causing shoulder pain, adhesive capsulitis, and pathologies involving arthritis of the shoulder joint must be considered in the differential diagnosis of SAIS [27].

Complications

Subacromial impingement syndrome can lead to several complications if not treated or poorly treated, such as degeneration and tearing of the rotator cuff, which has been reported in up to 65% of patients with longstanding SAIS. Adhesive capsulitis, affecting approximately 2% to 5% of the general population, is more prevalent among individuals with shoulder pathology and can result in prolonged stiffness and functional limitation [27]. Cuff tear arthropathy, though less common, typically arises in patients with massive rotator cuff tears and progressive joint degeneration. Complex regional pain syndrome, although rare, has been documented as a potential sequela following shoulder injury or surgery. These additions aim to provide a more evidence-based overview of SAIS-related complications.

Radiological modalities

Radiographs can show structural factors that cause impingement, like acromial spurs, sclerosis, cystic changes, or a narrowing of the acromiohumoral interval. There are three types of views: anteroposterior (AP), scapular Y, and supraspinatus outlet projections. MRI presents a clear picture of soft tissues and can identify bursitis, tendon problems, and thickening of ligaments. But MRIs are usually done with the arm at rest, so they might not fully show how impingement works [28].

Management (non-surgical and surgical)

Non-surgical Management

Conservative management is preferred according to current studies, which include [29]. Exercise and manual therapy enhance shoulder function and alleviate pain. Outcomes are better when combined with manual techniques (like joint mobilisation) than with exercise alone.

Non-steroidal anti-inflammatory drugs (NSAIDS), such as Oral diclofenac, are used clinically in SAIS to decrease inflammation and pain. However, researchers have demonstrated limited efficacy in enhancing functionality and return-to-work rates relative to corticosteroid injections, which provide more targeted anti-inflammatory effects in the subacromial space.

Shockwave therapy has been used as a treatment option for SAIS, aiming to stimulate tissue healing and reduce pain through acoustic waves. While some studies have explored its potential benefits, current evidence from randomized controlled trials remains inconclusive. For instance, a systematic review found that shockwave therapy did not demonstrate superior outcomes compared to placebo or standard physiotherapy in terms of pain reduction or functional improvement [29]. Variability in treatment protocols, energy levels, and patient selection across trials limits the consistency of findings. Given these limitations, shockwave therapy should be considered cautiously and not adopted as a primary management strategy. Instead, well-established physiotherapy programs to be the cornerstone of non-surgical treatment for SAIS.

Ultrasound and laser therapy have been used in the treatment of SAIS to relieve pain and promote tissue healing. However, Moderate-quality evidence suggests that both modalities provide negligible benefits beyond the placebo effect. As a result, these therapies are often considered supplementary rather than primary interventions.

Platelet-rich plasma (PRP) injections, in which concentrated platelets are injected to promote tissue healing, have been evaluated as a potential treatment for SAIS. Recent studies suggest that steroid injections reduce inflammation more compared to PRP in the short term. Due to these studies' findings, PRP’s role in SAIS management remains limited. Approximately 60-90% of patients with SAIS experience improvement with conservative treatment when diagnosed early. [27].

Surgical Management

Acromioplasty can be done with an arthroscope or through open surgery, reshaping the acromion to relieve pressure on the subacromial space. The acromioplasty aims to get a flat type 1 acromion to reduce damage to the tendon. Arthroscopic techniques provide expedited recovery and reduced short-term complications; however, long-term outcomes are comparable to open procedures [30].

## Conclusions

Subacromial impingement syndrome is a leading etiological factor of shoulder discomfort that can be challenging to manage effectively. It happens because of a mix of things, including internal problems (like wear and tear on the tendon from overuse or ageing), external problems (like compression from nearby structures), and biomechanical problems (like the shoulder moving in an unusual way). For many people with SAIS, treatments that don't involve surgery, such as physical therapy, exercise, or medication, usually work to ease pain and restore the body to normal. For people who don't see improvements with non-surgical methods, surgical options like debridement or acromioplasty (which can be done arthroscopically or through an open approach) can be advantageous. Research shows that both surgical and non-surgical treatments often work the same way when it comes to relieving pain and improving shoulder function.

Lastly, to refine diagnostic precision and optimize treatment pathways, future research should focus on high-quality randomized controlled trials comparing long-term outcomes, cost-effectiveness analyses of treatment modalities, and the development of advanced diagnostic tools such as dynamic imaging and validated clinical test clusters. These efforts are essential to improve patient stratification and guide evidence-based care for SAIS.
